# Alternative *in-vivo* models of mucormycosis

**DOI:** 10.3389/fcimb.2024.1343834

**Published:** 2024-02-01

**Authors:** Jakob Scheler, Ulrike Binder

**Affiliations:** Department of Hygiene, Microbiology and Public Health, Division of Hygiene and Medical Microbiology, Medical University Innsbruck, Innsbruck, Tirol, Austria

**Keywords:** mucormycosis, alternative model organisms, *Galleria mellonella*, *Drosophila melanogaster*, *Caenorhabditis elegans*, *Danio rerio*, *Bombyx mori*

## Abstract

Mucormycosis is still regarded a rare fungal infection, but the high incidences of COVID-associated cases in India and other countries have shown its potential threat to large patient cohorts. In addition, infections by these fast-growing fungi are often fatal and cause disfigurement, badly affecting patients’ lives. In advancing our understanding of pathogenicity factors involved in this disease, to enhance the diagnostic toolset and to evaluate novel treatment regimes, animal models are indispensable. As ethical and practical considerations typically favor the use of alternative model systems, this review provides an overview of alternative animal models employed for mucormycosis and discusses advantages and limitations of the respective model.

## 1 Introduction

**Figure 1:**
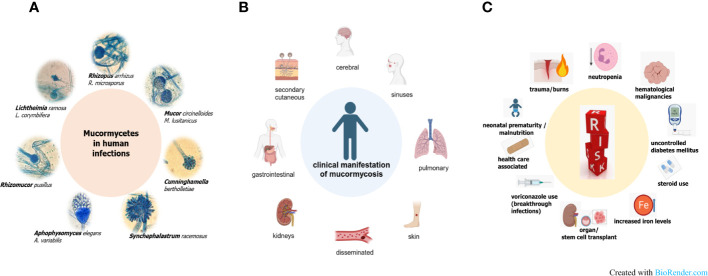
Pathogenic agents **(A)**, various forms of disease **(B)** and risk factors **(C)** in mucormycosis.

Mucormycosis is a severe, quickly progressing and highly necrotizing fungal infection, that is life-threatening particularly for immunodeficient patients (Gupta et al., 2023). There is one term that maybe describes this disease best, which is heterogeneity. This heterogeneity in the pathogens causing these infections, in the forms of disease and the repertoire of underlying diseases is associated with drawbacks in understanding mucormycosis (**Figure 1**). Among the Mucormycetes, an ancient and very diverse group of fungi, species belonging to the order Mucorales are involved in causing severe infections in human (Roden et al., 2005; Hoffmann et al., 2013). Worldwide, the majority of infections is attributed to the genera *Rhizopus*, *Lichtheimia* and *Mucor* (**Figure 1A**). Depending on geography and/or underlying disease, the species distribution varies and other genera such as *Apophysomyces*, *Saksenaea* — both often associated with cutaneous mucormycosis (Al-Zaydani et al., 2015; Sigera et al., 2018; Chander et al., 2021; Gupta et al., 2022; Planegger et al., 2022) — *Cunninghamella* or *Rhizomucor* are reported (Gomes et al., 2011; Raju et al., 2020; Hallur et al., 2021; Schober et al., 2021). Mucorales are ubiquitously found in the environment and share common features like the coenocytic thallus and a cell wall containing chitosan. On the other hand, this complex fungal group exhibit species-specific differences in important mechanisms such as invasion pathways, host defense mechanisms, cell wall and plasma membrane composition, spore size, prevalence of different spore types or yeast-hyphae dimorphism (Ribes et al., 2000; Richardson, 2009; Li et al., 2011). For example, *Rhizopus* species have been shown to be phagocytized by alveolar macrophages which leads to inhibition of germination, although the spores stay viable, whereas *Mucor* spores are able to germinate within the cell resulting in macrophage cell death (Voelz et al., 2015; Andrianaki et al., 2018; López-Muñoz et al., 2018).

The different forms of mucormycosis (**Figure 1B**) strongly reflect the route of infection of the pathogenic agent, the spores. Inhalation of spores results in rhino-orbital-cerebral forms or pulmonary forms, which comprise the highest percentage of mucormycosis cases. Species dominating clinical samples of these patients are naturally those that produce airborne spores, such as *Rhizopus* and *Rhizomucor* spp. Species of the genus *Mucor* or *Lichtheimia* produce wet sporangia that are set free in droplets This fact could explain why these fungi are more often associated with major trauma, including burn wounds (Walther et al., 2019; Gupta et al., 2023). In these cutaneous infections, transmission takes place via direct contact with contaminated material. This can be wooden splints, as it has been observed in patients exposed to natural disasters like tsunamis or tornadoes, but also clinical material such as contaminated bandages. Rarely, even very small traumata such as animal scratches or insect bites have been the entry portal for spores in patients developing a cutaneous form of disease (Skiada et al., 2022). Ingestion of contaminated foods or drinks has been described as the reason for gastrointestinal mucormycosis, which is more prevalent in Asia than in other continents. All forms of disease share a fulminant progression due to fast, abundant growth, angioinvasion, which facilitates dissemination of the disease, and extensive tissue necrosis. The high likelihood of dissemination together with the limited treatment options explain a higher percentage of secondary cutaneous forms when compared to other, non-mucormycete fungal infections (Cornely et al., 2019; Skiada et al., 2020; Clark et al., 2023). Predisposing factors are in part the same as for other invasive fungal infections (**Figure 1C**): mainly neutropenia, followed by hematological malignancies, organ, and stem cell transplantation or long-term corticosteroid use. Other risk factors, more prominent in mucormycosis than in other infections, comprise diabetic ketoacidosis in poorly controlled diabetic mellitus patients or iron overload. Elevated iron levels by deferoxamine therapy, traumata or use of ineffective antifungals such as voriconazole leading to breakthrough infections are additional factors putting patients at risk. Also, health care associated cases, in which spores were transmitted via contaminated material like mouth spatulas or bandages have been reported, as well as mucormycosis (often gastrointestinal) in neonates or malnourished patients (Jeong et al., 2019; Sharma et al., 2023). Diabetes was shown to be an underlying disease in one third of mucormycosis patients in the so far biggest retrospective study comprising more than 900 patients (Roden et al., 2005). A more recent survey from Greece that included data from 108 cases found a lower percentage (15.9%) attributed to diabetes. Interestingly, the same study showed, that more than 20% of the cases involved immunocompetent persons that suffered from cutaneous or soft tissue infections after traumatic events (Drogari-Apiranthitou et al., 2023). The mucormycosis outbreak in India among COVID-19 patients, which led to 40 000 CAM (COVID-associated mucormycosis) cases, has shown that an interplay of several host and iatrogenic factors led to the high emergence of CAM. Prakash and co-authors have extensively reviewed factors leading to the CAM outbreak (Prakash et al., 2021). Hyperglycemia, either because of pre-existing diabetes mellitus or dysfunctional insulin production because of virus-damaged pancreatic cells, or as a consequence of corticosteroid therapy, were found to be the main risk factors. Additionally, as known in diabetic ketoacidosis, many patients showed high ferritin levels, consequently leading to impaired iron homeostasis. Irritation in iron homeostasis and higher free iron levels were enhanced by the viral infection, which causes hemoglobin to release iron (Prakash et al., 2021). A recent meta-analysis comparing data of 958 CAM cases from 45 countries, which were mainly middle and low-income countries, also identified corticosteroid use and diabetes as the major risk factors. Additionally, older age, an *Aspergillus* coinfection or the use of tocilizumab were determined as players in causing CAM (Özbek et al., 2023).

Whether mucormycosis is associated with COVID-19 or not, the therapeutic problem is the same. Mucormycetes spread very fast in host tissue and their angioinvasive character ultimately causes thrombosis and tissue necrosis, which makes surgery in addition to antifungal treatment necessary in many cases. Unfortunately, this group of fungi exhibits intrinsic resistance to commonly used antifungal drugs like short-tailed azoles and elevated minimum inhibitory concentration (MICs) against polyenes are common as well, resulted in unacceptable high mortality rates even with treatment (Cornely et al., 2019). In order to develop novel treatment strategies, a better understanding of the disease is essential. Especially in mucormycosis, where, except for CAM cases in India, the patient number is low and therefore patient samples are rarely available and clinical trials are also limited by the great heterogeneity, animal models are of great importance. Obviously, mammalian models are considered the gold standard and many have been established for studying mucormycosis. Jacobsen has given a comprehensive review on that topic recently explaining infection routes, mimicking of the various forms of disease and discussing advantages and limitations (Jacobsen, 2019). Practical difficulties and ethical restrictions put a limit to the use of mammalian models for many laboratories. Furthermore, high throughput screenings or comparison of a large strain set is impossible. Especially in the case of mucormycosis, representing great heterogeneity in the species involved or the antifungal resistance profile, such high throughput assays are immensely important. Therefore, this review will focus on alternative model systems that have been exploited so far for mucormycosis studies, highlighting novel alternative model animals or adapted methods established in the last three to four years to avoid repetition of what has already been reviewed in detail by Jacobsen (2019). The alternative animal models presented in the review have significantly contributed to fostering our understanding of previously undescribed virulence factors of Mucorales, some of which are unique to this group of fungi (Patiño-Medina et al., 2018; Patiño-Medina et al., 2019a; Wurster et al., 2020; Alejandre-Castañeda et al., 2022; Itabangi et al., 2022; Trieu et al., 2022; Szebenyi et al., 2023). Moreover, these models have unveiled pathogen recognition and immune evasion strategies of Mucoralean fungi that are relevant for interactions with the human host (Chamilos et al., 2008a; Hoffmann et al., 2013; Inglesfield et al., 2018; López-Muñoz et al., 2018; Hassan et al., 2021; Itabangi et al., 2022).

## 2 General aspects of alternative models

What are the expectations of an alternative animal model? Independent of the infection, alternative hosts are expected to be easily obtained, either by breeding or by commercial availability in high numbers, easy to handle by the operators and less cost intensive than mammalian models, which allows for high throughput screening. Good correlation in the outcome to mammalian models are a prerequisite and ideally, different routes of infection and different forms of disease can be investigated. The use of alternative infection models is in accordance with the 3R policy of replacement, reduction, and refinement of animal utilization in research (Tannenbaum and Bennett, 2015). Over the years, many alternative hosts have been used and optimized to study pathogenicity factors or efficacy of antifungal treatment. In the following chapters, the principles of the individual model and recent findings obtained with each are summarized.

## 3 Invertebrate models

### 3.1 Caenorhabditis elegans

#### 3.1.1 General aspects, advantages and disadvantages

The nematode *C. elegans* occurs commonly in soil, decaying plant matter as well as inside the gut of some invertebrates. These environments are naturally abundant in microbes, representing also the main food source that *C. elegans* feeds on. In the laboratory setting, worms are cultivated on agar plates or in liquid culture with *Escherichia coli* OP50 as their nutritional source. This bacterial strain is a biofilm formation defective mutant and uracil auxotroph (Arata et al., 2020). The worms are typically maintained at temperatures ranging from 15 to 25°C (Schulenburg and Félix, 2017). The advantages of using this model organism, relevant for testing all kinds of fungi, are its small size, transparent body, short life cycle and cost-effectiveness in large-scale cultivation. Furthermore, its genome is completely sequenced and well annotated and its genetic manipulation, particularly through RNA interference, is well established (Fire et al., 1998; Lamitina, 2006). Additionally, machine learning solutions enable automated high-throughput screenings to evaluate worm survival following infection with pathogens or when testing the toxicity and efficacy of antimicrobial agents (Wählby et al., 2012). In these screenings, the shape of *C. elegans* in liquid culture, as observed in bright field microscopy images, is assessed, with sinus-shaped worms considered being alive and straight ones considered dead (Marsh and May, 2012; Wählby et al., 2012; Singulani et al., 2018).

However, when studying fungal infections, *C. elegans* presents two main disadvantages. First, due to their small size (with adult animals averaging at around 1 mm in length), the worms cannot be infected with a defined inoculum size. Instead, *E. coli* OP50 is simply replaced with the relevant infective agent as the food source for *C. elegans* (Muhammed et al., 2012). Additionally, *C. elegans* worms cannot withstand temperatures higher than 25°C for extended periods. For studying virulence factors of fungal pathogens *in-vivo*, it is crucial to mimic human host temperatures if aiming to mimic invasive forms of disease. This temperature constraint can be a limitation when studying certain virulence factors, such as temperature-dependent morphological switches (Klein and Tebbets, 2007; Marsh and May, 2012).

While *C. elegans* has been previously employed as a model for studying fungal infections, as comprehensively reviewed by Arvanitis et al. (2013); Fusco-Almeida et al. (2023); Singulani et al. (2018); Jacobsen (2019) noted its absence in studying mucormycosis. Recent publications from the group of Víctor Meza-Carmen at Universidad Michoacana de San Nicolás de Hidalgo adopted the model for investigating the virulence of *Mucor circinelloides* (now classified as *M. lusitanicus)* knockout strains as well as the toxicity of secreted metabolites in their respective cell-free supernatants.

#### 3.1.2 The use of *C. elegans* for studying virulence potential

Patiño-Medina et al. utilized the model to study the role of ADP-ribosylation factors (Arf) in the virulence of *M. lusitanicus*, demonstrating increased virulence potential in both the knockout strains and their supernatants (Patiño-Medina et al., 2018). Similarly, the group used the model animals to investigate the role of Arf-like proteins (Arl1 and Arl2), that are involved in vesicle trafficking and tubulin assembly, in virulence of the same fungus. They observed higher virulence of sporangiospores of arl1 and arl2 deletion strains and higher virulence of their cell-free supernatants when incubating them with *C. elegans* (Patiño-Medina et al., 2019a). According to the authors, these results collectively suggest that harmful proteins secreted by the fungus are responsible for increased virulence, also in the supernatant. However, the exact protein(s) in question still need to be identified. In another investigation employing the same approach, Díaz-Pérez et al. explored the fermentative metabolism of *M. lusitanicus* and linked increased levels of secreted acetaldehyde in the knockout strain to enhanced virulence in the nematode model (Díaz-Pérez et al., 2020). Similarly, the group investigated the role of siderophores, specifically rhizoferrin, in the virulence of *M. lusitanicus*. As reported by Alejandre-Castañeda et al., the deletion of the rhizoferrin encoding gene (*rfs*) resulted in decreased virulence of the cell-free supernatant and reduced virulence of the sporangiospores compared to the wild type (Alejandre-Castañeda et al., 2022). In 2019 and 2021, Patiño-Medina et al. conducted experiments to determine whether the medium on which sporangiospores of *M. lusitanicus* are produced would influence their virulence (Patiño-Medina et al., 2019b; Patiño-Medina et al., 2021). When yeast extract-peptone-glucose (YPG) medium was supplemented with native blood serum, the virulence of the sporangiospores increased, leading to an elevated mortality rate of *C. elegans* when compared to sporangiospores grown on standard YPG (Patiño-Medina et al., 2019b). Additionally, they demonstrated that sporangiospores produced from rice showed similar rates of virulence against *C. elegans*. Using rice as a growth medium allowed for more cost-effective mass production of sporangiospores compared to YPG-medium (Patiño-Medina et al., 2021).

In 2022, Richter et al. utilized *C. elegans* and another nematode, *Aphelenchus avenae*, to investigate the potential nematodocidial effects of the toxin rhizoxin, produced by endosymbiotic bacteria of *Rhizopus microsporus* (Richter et al., 2022). Their study revealed that the secondary metabolite exhibits toxic effects towards both organisms. Furthermore, they showed that *A. avenae*, actively feeds on endosymbiont-free *R. microsporus* whereas endosymbiont-harboring fungus is lethal towards the nematode through starvation or exposure to the toxin.

### 3.2 Drosophila melanogaster

#### 3.2.1 General aspects, advantages and disadvantages

The fruit fly, *D. melanogaster*, has been a pivotal and intensively studied model organism in biological research since the beginning of the 20^th^ century. Originally hailing from tropical regions, *D. melanogaster* has expanded its habitat to temperate zones. In the wild, these flies feed on fermenting fruits, where they engage in mating and lay their eggs. Possessing a highly conserved innate immune system, *D. melanogaster* facilitates the transferability of experimental findings to mammalian hosts (Younes et al., 2020; Michael Harnish et al., 2021). Moreover, the utilization of a broad number of genetic tools for genome manipulation has rendered it a highly tractable and precisely manipulable model organism, aiding in uncovering the functions of conserved genes in the human genome and deciphering recognition of fungal infections via the Toll-pathway (Lemaitre et al., 1996; Bellen and Yamamoto, 2015; Troha and Buchon, 2019).

One of the main limitations of the model is the limited tolerability of temperatures higher than 32°C for the completion of its life cycle, resulting in experiments usually performed at 29°C (Schou et al., 2017; Wurster et al., 2019a). Additionally, due to its diminutive size (with adult female flies measuring around 3 mm), infecting them with a defined inoculum requires a microinjector. For the study of fungal infections, anesthetized adult flies are pricked with a stainless steel needle dipped into a defined fungal spore solution or exposed directly to the sporulating fungus on its culture plate (Troha and Buchon, 2019).

In studying mucormycosis, adult *D. melanogaster* flies have been employed to show the significance of pathogen recognition for the development of infection. One of the breakthrough understanding in immunology against Mucorales was by a study in *Drosophila*, which highlighted significant difference in recognition and host response between *R. oryzae* and *A. fumigatus*. Phagocytosis of *R. oryzae* spores and damage to hyphae was shown to be a lot lower than to *A. fumigatus*, which explained the establishment of invasive disease even in immunocompetent flies, while these would be resistant to infection with *A. fumigatus* (Chamilos et al., 2008a; Chamilos et al., 2008b). Further, flies have been utilized to explore novel antifungal approaches (Chamilos et al., 2006; Lewis et al., 2013; Mircus et al., 2015; Bellanger et al., 2016; Ben Yaakov et al., 2016), to investigate the effects of pretreatment with azoles (Lamaris et al., 2009; Wurster et al., 2019b) and to examine the influence of fungal iron metabolism on virulence (Chamilos et al., 2008a; Pongas et al., 2009). As these works have been extensively reviewed by Jacobsen (2019), the focus here is on more recent publications.

#### 3.2.2 The use of *D. melanogaster* for studying virulence factors

Additionally, the fly model is used to study genetically modified Mucorales strains. Ibragimova et al. created uracil auxotrophic strains of *Lichtheimia corymbifera* by disrupting the pyrG-gene via CRISPR-Cas9 and evaluated their pathogenicity in comparison to the original strain in the *D. melangoster* model (Ibragimova et al., 2020). Szebenyi et al. investigated the role of spore coat proteins (CotH) in virulence of *M. lusitanicus* by employing the *D. melanogaster* model as well as the *G. melonella* and the diabetic ketoacidosis mouse model (Szebenyi et al., 2023). They demonstrated that mutants with CRISPR-Cas9 mediated gene disruptions in CotH3 or CotH4 protein encoding genes exhibited reduced virulence in the mouse and fly models, but only for CotH4 in *G. mellonella*. Both proteins are important for temperature adaptation and cell wall development. CotH4 additionally plays a role in spore wall formation.

Exploring the impact of tornadic shear stress as exerted on spores during natural catastrophes like tornados, Wurster et al. compared the influence of such mechanical stress events on the virulence of different mold species (Wurster et al., 2020). Employing the *D. melanogaster* model, they observed a transient hypervirulence only in species belonging to the order Mucorales. After ruling out an interfering influence of shear-stress challenged spores of *R. arrizhus* on the immune capacity of the host’s phagocytic cells, the authors demonstrated that the increased virulence of stressed spores resulted from soluble factors secreted by the fungus. The inhibition of hypervirulence by adding calcineurin inhibitors during or after shear-stress exposure highlighted the pivotal role this pathway plays in pathogenicity. The pathway’s relevance was further underlined by exposing *M. lusitanicus (former M. circinelloides f. lusitanicus)* strains with loss-of-function mutations in genes responsible for calcineurin protein synthesis to shear-stress. In these strains, shear-stress led to no significant higher virulence, due to the inability of these strains to synthesize calcineurin. Lee et al. previously demonstrated the avirulence of the regulatory B subunit deletion strain in *G. mellonella* model (Lee et al., 2013). Additionally, Vellanki et al. demonstrated that when exposing macrophages to calcineurin lacking mutants phagosome maturation was higher compared to the wild type, underlining the importance of calcineurin to support immune evasion (Vellanki et al., 2020). Conversely, their ability to cause damage to endothelial cells was lower when compared to the wild type.

#### 3.2.3 Translational aspects

The *Drosophila* model has been used to study the detrimental toxic effects of volatile organic compounds (VOCs), a possible contributing factor to fungal pathogenicity (Bennett and Inamdar, 2015; Zhao et al., 2017). Macedo et al. demonstrated that 1-octen-3-ol decreased survival and inhibition of locomotion in adult *D. melanogaster* flies due to toxic effects of the compound on mitochondria, causing inflammation and apoptosis (Rosowski et al., 2018). Almaliki et al. cultured different medically relevant pathogenic fungi (excluding Mucorales) together with instar larvae of *D. melanogaster* in a microhabitat, exposing them to fungal VOCs but not directly to the fungi (Almaliki et al., 2020). They observed development delays in insect metamorphosis when exposed to pathogenic strains producing high concentrations of VOCs, such as1-octen-3-ol. Conversely, Zhao et al. found, that although *Mucor racemosus* emitted the highest amount of VOCs among the tested fungi, it did not significantly influence the mortality of 3^rd^ instar larvae (Zhao et al., 2017). This could possibly be linked to the low amount of 1-octen-3-ol *M. racemosus* produced in comparison to the more toxic *Aspergillus* strains that exhibited higher secretion of this VOC. While the profiling of VOCs from patient’s breath may provide a new tool for noninvasive diagnostics of fungal diseases (Acharige et al., 2018; Licht and Grasemann, 2020), there is still a lack of research on the role of VOCs in the pathogenicity of Mucoralean species and their specific VOC profile for diagnosis. A study as the one carried out by Li et al., investigating breath samples of 53 patients with chronic pulmonary aspergillosis, are of great importance (Li et al., 2021). For pulmonary mucormycosis, is will be a great challenge to collect a similar number of samples due to the high variability of pathogens causing the disease. Here, alternative model systems, like the already employed *Drosophila* model, will significantly contribute in the development of such VOC based diagnostics tools.

### 3.3 Galleria mellonella larvae

#### 3.3.1 General aspects, advantages and disadvantages

*G. mellonella* (the Greater Wax moth) is a member of the order *Lepidoptera* and the family *Pyralidae*, which are found globally. Naturally, the larvae are a pest of bee hives, and they are bred as bait for fishing or reptile food (Jafari et al., 2010). Over the last 20 years, the larvae gained an important role as model host to study microbial infections. This model has been employed globally for dozens of fungal species, including species of the Mucorales (Curtis et al., 2022). The structural and functional similarities of the insect immunity and the innate immune system of mammals help to gain comparable results to murine models. Fungi are recognized via pathogen-associated patterns (PAMPS) which are homologous to those in mammals, and both a humoral and cellular response is activated. Six different types of hemocytes have been described circulating in *Galleria* hemolymph that orchestrate to prevent fungal growth by phagocytosis, nodulation, agglutination, encapsulation, and secretion of antifungal peptides (Trevijano-Contador and Zaragoza, 2019). It has been shown by the Kavanagh group that hemocytes have a comparable mechanism to the NADPH oxidase complex of human neutrophils, and produce superoxide and their activity could be diminished by mycotoxins such as gliotoxin or fumagillin (Bergin et al., 2005; Fallon et al., 2011). The reasonable size of the larvae makes it easy to inoculate a defined amount of spores, which in most protocols is done via the prolegs, which provide a natural entrance to the body, and a thin needle can enter without harming the animal. Importantly, *Galleria* larvae can be used over a wide temperature range from 20-42°C, so they are ideal to mimic human body temperature (Fuchs et al., 2010). The larvae can be obtained commercially or easily bred in an incubator if only small numbers are needed. This, and the low costs, are big advantages and facilitate high throughput screenings. Unfortunately, the different origin of larvae represent one of the main limitations of this model, rendering the comparison of results between laboratories difficult.

Results are obtained rapidly and a number of experimental endpoints and readouts are possible, which have been summarized in details by Curtis et al. (2022). Larval survival, histopathological examination, determination of fungal burden and defining the rate of melanization are the most commonly applied readouts, either to compare virulence potential of a high number of strains or to evaluate efficacy of antifungal drugs or combination thereof. With all the advantages and methods developed for Mucorales in *Galleria* larvae, only a higher standardization of protocols is needed to make intra-laboratory comparisons easier. For example, a striking point, when comparing the methods sections of individual manuscripts, is the great variability in inoculum size used even for the same strain. Ranges differ between 2×10^3^ spores – 10^7^ spores per larva, leading to more or less the same mortality rates. Reasons thereof comprise the different larval batches that are used (in which larval size can vary a lot), potential differences in growth medium to obtain the spores solutions, and in inoculum preparation, where no standardized protocols are yet defined for filtration of spore solutions, storage, or freezing temperatures. Having comparable data and deciphering the impact of variabilities in these simple methodological aspects might improve the quality of data obtained from *Galleria* experiments in the future.

The larval model has also been used frequently for studying mucormycetes. Pioneering works have been reviewed in detail by Jacobsen already (Jacobsen, 2019). They comprise investigations of inter-and intraspecies dependent virulence potential, correlating differences in temperature adaptation, spores size, growth speed, oxidative stress tolerance, or if higher iron concentrations in the growth medium affect virulence of spores (Schwartze et al., 2012; Kaerger et al., 2015; Maurer M. et al., 2019).

#### 3.3.2 The use of *G. mellonella* for studying virulence factors

A recent study investigating the thermal adaptation of *Mucor irregularis*, once more, demonstrated that strains growing well in high temperature also cause higher mortality rates in infected larvae, which was underlined by higher fungal burden determined by fluorescent staining of homogenized dead larvae (Zhang et al., 2022). Deciphering virulence traits on a genetic level has been delayed in Mucorales due to unavailability of annotated genome sequences and their reluctance to genetic modification. Luckily, this has improved tremendously in the last decade and, although it is still more difficult than in other fungi, great tools and protocols have been established to delete or silence genes (Lax et al., 2022). To decipher the role of these genes for virulence, *Galleria* larvae have been employed. RNAi silenced mutants of *M. circinelloides f. lusitanicus* (now *M. lusitanicus*) showed that phospholipaseD and a myosin V family transporter are necessary to establish full virulence in both, *Galleria* larvae and mice (Trieu et al., 2017). The same group recently demonstrated that myosin II plays an important role in growth, sporulation and hyphal development in *Mucor.* Interestingly, only disruption of one myosin II gene, *mcmyo2A*, led to abrogation of pathogenicity, while *mcmyo2B* deletion had no impact on virulence, although the mutant was similarly attenuated in growth (Trieu et al., 2022). Iron acquisition is crucial for pathogenicity also in Mucorales, as *M. lusitanicus* that is not able to produce rhizoferrin, the mucormycete siderophore, was shown to be avirulent. Moreover, increased virulence was observed for the same species when spores were generated on serum containing agar plates which goes in hand with higher mitochondrial activity (Patiño-Medina et al., 2023).

#### 3.3.3 Immunological aspects investigated by the use of Galleria larvae

Two studies investigated the role of the spore surface layers or proteins in the recognition process, effect on phagocytosis leading consequently to changes in virulence potential. In *Lichtheima corymbifera*, spores were physically and chemically modified to get rid of one cell wall layer after the other to determining the influence of each layer in recognition and on the spore uptake by macrophages. Surface modification led to an increased rate of phagocytosis especially in those confronted with glucanolytic enzymes or pronase E. In *Galleria* larvae a combination of the latter led to attenuated virulence potential (Hassan et al., 2021). A study deciphering the role of cotH genes in *M. lusitanicus*, showed comparable data in larvae and a diabetic ketoacidosis (DKA) mouse model. Of the five cotH genes that were deleted (*Mucor* has 17 cotH genes altogether), two deletions, cotH3 and cotH4 caused attenuated virulence compared to the wild type strains. Contrary to *Lichtheimia*, where changes in the proteins of spore surfaces rendered them easier to phagocytize, *Mucor* lacking CotH proteins were not affected in their uptake by macrophages, nor was the acidification of phagosomes impaired (Szebenyi et al., 2023).

#### 3.3.4 The use of *G. mellonella* for studying antifungal efficacies

*Galleria* larvae were also employed to test antifungal treatment efficacy against mucorales species. The first such study by Maurer et al. investigated efficacy of a single dose of azoles and polyenes, including a modified formulation of nystatin, nystatin-IL, and isavuconazole. The single application of nystatin-intralipid exhibited the best activity against Mucorales, followed by posaconazole, while limited efficacy was seen for liposomal amphotericin B and isavuconazole in larvae. Furthermore, the effect on hemocyte density; was evaluated to rule out an unspecific immune response caused by the drug. Importantly, pharmacokinetics of antifungals can, and probably should be examined before concluding (in)efficacy of drugs. Maurer et al. showed a linear increase in C_mas_ and AUC_0-24_ in larval haemolymph which is similar to human serum, but differences emerged regarding metabolism and half-life. These parameters are relatively easy to study in the larvae and would contribute to a better comparison between larval and mammalian models (Astvad et al., 2017; Maurer E. et al., 2019). Recently, combinatorial treatment regimen of voriconazole and either amphotericin B, posaconazole or caspofungin were evaluated in *Galleria* larvae increasing larval survival in comparison to the single treatments. Of note, multiple injections at 3 time points (2 h, 26 h and 50 h post infection) of the antifungals drugs were carried out and no negative effect on the larvae by the multiple injections was observed (Macedo et al., 2019). One of the limitations of *in vitro* antifungal susceptibility testing is, that in standardized protocols, drug efficacy is tested against spores, a morphology rarely found on the site of infection. Lately, a single dose of amphotericin B (10 mg/kg) against spores or germlings of *R. arrhizus* and *L. corymbifera* was shown to be efficient only against infections with spores but not germlings, where all larvae died 24 h after infection, independently of receiving amphotericin B or not (Samdavid Thanapaul et al., 2023).

Attempts to facilitate and speed up *in-vivo* antifungal susceptibility testing have been made for *Candida* and *Aspergillus* species, in which luciferase reporter strains were used for *in-vivo* detections of infections and monitoring of drug efficacy in either living larvae or the homogenized pulp of the infected animal (Delarze et al., 2015; Vanhoffelen et al., 2023). In Mucorales luciferase expressing strains have been generated in *M. lusitanicus*, but light emission was shown to be too weak for animal experiments in strains with non-codon optimized luciferase. Luciferase expressing strains, once codon optimized, will not only facilitate detecting fungal infections but could also shed light on *in-vivo* transcriptional regulation of virulence relevant genes in the future. Moreover, *Galleria* larvae definitely represent a very useful tool for prescreening assays enabling the reduction of mouse studies needed for confirmation.

### 3.4 Bombyx mori larvae

#### 3.4.1 General advantages and disadvantages

The silkworm, *Bombyx mori*, stands as a domesticated insect species that has played a vital role in human economies for more than 5000 years (Xu and O’Brochta, 2015). Similar to other invertebrate model organisms, the silkworm provides distinct advantages in life science research due to its easy propagation, small size and manageable maintenance. Additionally, extensive genomic information has been scrutinized and a myriad of tools for genetic manipulation have been successfully established (Toshiki et al., 2000; Xia et al., 2008; Wei et al., 2014; Xu and O’Brochta, 2015; Baci et al., 2022). The silkworm, like other invertebrates, depends solely on its innate immune response, encompassing mechanisms such as melanization, phagocytic cells, antimicrobial peptides and lysozymes, to combat microbial infections. Detailed reviews on the immune strategies of this model organism can be found in works by Chen & Lu (Chen and Lu, 2018) and Kausar et al. (2019). In the realm of infection biology, precise concentrations of infectious agents can be administered via injection into the hemolymph or into the midgut of the 5th instar larvae (Meng et al., 2017). This offers an advantage in contrast to the *G. mellonella* model where infections are usually injected into the hemolymph without any organs specifically addressed (Matsumoto and Sekimizu, 2019). Due to the convenient size, organ extraction is feasible, enabling the study of dissemination of spores from the hemolymph (or midgut) to various tissues types (Yu et al., 2021). The silkworm has been established for studying bacterial infections as well as number of medical relevant fungal species like *Candida glabrata*, *Crpytococus neoformans* and *Aspergillus fumigatus* (Matsumoto and Sekimizu, 2019).

#### 3.4.2 The use of *B. mori* for studying antifungals and screening virulence potential of strains

Moreover, Yu et al. devised an easy-to-use protocol for visualizing diseases progression of filamentous fungal infection and evaluating treatment results with antifungal agents (Yu et al., 2021). Post-infection with *A. fumigatus* and *L. corymbifera*, they dissected and stained larval organs with calcofluor white for the visualization of fungal growth and microscopic examination. By optimizing their protocol, the authors could raise the maximum temperature for larval incubation to 34°C, reassembling conditions closer to those in the human host. Tominaga et al. utilized the silkworm infection assay to assess the dose-dependent mortality of infection with *R. oryzae* (Tominaga et al., 2018). Additionally, they tested the efficacy of amphotericin B, as well as new, potentially antifungal, compounds with moderate efficacies isolated from a hot spring-inhabiting *Penicillium* sp. in the *in-vivo* model. Similarly, Kurakado et al. evaluated the efficacy of posaconzaole against eleven *R. oryzae* isolates of different origins and examined variations in mortality rates based on incubation temperature and inoculum size (Kurakado et al., 2021). By reducing the inoculum size, they increased the incubation temperature to 37°C and successfully demonstrated posaconzaole’s therapeutic effect in the silkworm model. In addition, Panthee et al. isolated novel pathogenic strains belonging to *Mucor* and *Backusella* spp from plant biomass (Panthee et al., 2021). They demonstrated their pathogenicity in immunocompromised mice and the silkworm model, expanding the understanding of these strains’ virulence.

### 3.5 Mealworm (*Tenebrio molitor*)

Finally, Canteri de Souza et al. introduced the mealworm, *Tenebrio molitor*, as an additional host for investigating fungal infections (de Souza et al., 2015). Notably, this model can be maintained at temperatures up to 37°C. Fusco-Almeida et al. offer a comprehensive review of its applications for medically relevant fungi, however, as of yet, it has not been applied for the study of mucormycosis (Fusco-Almeida et al., 2023).

## 4 Vertebrate models

### 4.1 Zebrafish (Danio rerio)

#### 4.1.1 General aspects, advantages and disadvantages

The zebrafish, *D. rerio*, is naturally occurring in the river basins of the northeastern Indian subcontinent and stands as one of the most widely employed model organisms in biological research. Its small size, robustness, excellent reproductive performance and rapid development render it easy and cost-effective for laboratory maintenance and experimentation (Spence et al., 2008). With detailed genomic information, well-established tools for genetic manipulation, transparency of the larvae, allowing observation through live microscopy as well as its closer resemblance to humans compared to invertebrate models make it a strong model for the study of infectious diseases (Howe et al., 2013; Rosowski et al., 2018). Both larvae and adult fish are employed for these investigations, with larvae being particularly suitable for exploring the innate immune response due to the absence of an adaptive immune system (Rosowski et al., 2018). Regardless of differences in anatomy, the zebrafish can be used to study infections occurring in humans by injecting analogous organs with defined inocula (Gomes and Mostowy, 2020). One major disadvantage of this model, compared to invertebrates, is the need for specialized equipment for laboratory maintenance (Jacobsen, 2019). Moreover, even though wild zebrafish are able to tolerate temperature ranges from 10 to 38°C, domestication of zebrafish led to a reduced physiological plasticity due to their constant maintenance at their optimal temperature around 28°C (Morgan et al., 2022).

#### 4.1.2 The use of Danio rerio for studying the interplay of immune responses and virulence factors

For the first time employing the zebrafish model for the study of mucormycosis, Voelz et al. conducted experiments using the larval model to analyze the real-time-interaction between innate immune cells and *M. lusitanicus (formerly M. circinelloides f. lusitanicus)* sporangiospores *in-vivo* (Voelz et al., 2015). They could show that severity of the infection depended on infection site and was more pronounced and caused death sooner under dexamethasone-induced immunosuppression. In a follow-up study that combined zebrafish larvae with mathematical modelling, Inglesfield et al. could show that commencement of the disease depended on the number of recruited phagocytes in-*silico* (Inglesfield et al., 2018). These results were backed by the absence of spore killing and reactive oxygen burst *in-vivo.* Furthermore, they demonstrated that formation of granulomas is crucial for the control of fungal growth, which could recrudesce through immunosuppression. Building up on these previous studies, Itabangi et al. investigated the impact of endosymbiotic bacteria, specifically *Ralstonia picketti*, in *R. microsporus* on virulence and immune evasion (Itabangi et al., 2022). The authors demonstrated that bacteria free spores were avirulent compared to bacteria harboring wild-type spores in zebrafish larvae. For symbiont-free spores, the effect was independent of inoculum size and their morphological state (pre-swollen or resting). In contrast, endosymbiont-harboring spores showed higher virulence when pre-swollen. Additionally, the authors employed transgenic zebrafish larvae with fluorescently labeled phagocytes to examine macrophage and neutrophil recruitment. They demonstrated that the recruitment of both cell types was significantly lower in zebrafish larvae infected with endosymbiont-containing spores. The authors regarded the lower recruitment of immune cells as a form of immune evasion possibly mediated through secreted metabolites by the bacteria or their influence on the biological traits of the fungus, such as cell wall composition. Furthermore, they hypothesized that endosymbiont supported immunevasion could provide a possible explanation for cases of mucormycosis in immunocompetent patients (Hammami et al., 2021; He et al., 2021). Earlier work, examining the influence of a different endosymbiont of *R. microsporus*, namely *Burkholderia* spp., showed contradictory results, as no link between the presence of *Burkholderia* spp. as an endosymbiont and fungal virulence could be established. These studies highlight the species specific interactions of bacterial-fungal symbiosis in virulence (Ibrahim et al., 2008).

Kousser et al. revealed that co-culturing of *R. microsporus* and *Pseudomonas aeruginosa* lead to inhibition of fungal growth through the bacteria’s secretion of siderophores, specifically pyoverdine (Kousser et al., 2019). They further demonstrated that administering this siderophore would reduce the virulence of *R. microsporus* through iron starvation in the zebrafish larvae model compared to a siderophore that the fungus is able to to take up.

Expanded up on previous findings related to phagocyte recruitment, López-Muñoz et al. showed that an infection with sporangiospores of *M. lusitanicus* lead to a decrease in myeloid cells in the hematopoietic organ of the adult zebrafish, indicating their mobilization to the site of infection (López-Muñoz et al., 2018). Additionally, they demonstrated that the fungus could evade immunity though inducing cell-death in the macrophages but not in neutrophils, emphasizing the importance of neutrophils in protection from fungal diseases and the vulnerability of patients with neutropenia (Ghuman and Voelz, 2017). Furthermore, the authors demonstrated that a larger size of sporangiospores constitute a factor leading to higher virulence of the fungus.

Employing the adult zebrafish as a model to examine the effects of an infection by *Rhizopus oryzae*, with and without immunosuppression, Tatara et al. did not observe a statistical significant difference between the two trial groups (Tatara et al., 2018). These results contradict earlier observations made by Voelz et al. for the zebrafish larvae, yet no specific hypothesis could be found for this phenomenon (Voelz et al., 2015).

### 4.2 Embryonated chicken (Gallus domesticus) egg

#### 4.2.1 General aspects, advantages and disadvantages

A little more than 10 years ago, fertilized chicken eggs have been employed as one alternative vertebrate model to study fungal pathogenicity. The fertilized eggs provide the expected advantages like low costs, possibility of high throughput screening, facile maintenance and, importantly, are incubated at 37°C. Furthermore, administration of the pathogenic agent is possible via different routes (Jacobsen et al., 2012).

#### 4.2.2 The use of embryonated chicken egg for studying virulence factors

So far, the model has only been used in two works involving clinically relevant mucormycetes. They have shown to be a valuable tool for comparing the virulence potential of a high number of strains. Evaluating the virulence potential in *Rhizopus* and its correlation to temperature, stress resistance and morphological characteristics Kaerger et al. showed that adaptation to host temperature is not the single factor determining pathogenicity in comparison to species with less tolerability to 37°C (Kaerger et al., 2015). Similarly, different virulence potential was determined for species of the genus *Lichtheimia* which reflect the species isolated from patient material (Schwartze et al., 2012). To our best knowledge, no other studies including embryonated chicken eggs to study mucormycosis relevant mechanisms have been published. Even though, this model could also be used for testing antifungal efficacy and toxicity of novel drugs (Ribeiro et al., 2022).

## 5 Other

According to the ‘Amoeboid Predator-Fungal Animal Virulence’ Hypothesis, amoeba predation on fungi leads to evolutionary selection of fungal traits being advantageous for infecting animal hosts. These traits not only protect fungi from the host immune system but also influence interactions with phagocytizing amoeba (Casadevall et al., 2019). The evasion of host phagocytosis by certain fungal species like *Cryptococcus neoformans* to evade mediated host immunity has been investigated using amoeba, such as *Acanthamoeba castellani* or *Dictyostelium discoideum* (Steenbergen et al., 2001). Using the *Dictyostelium* model for phagocytosis, Itabangi et al. demonstrated that *R. microsporus*, in symbiosis with a metabolite secreting bacterial endosymbiont, suppressed the amoeba growth and inhibited their predation on other microbes (Itabangi et al., 2022). These findings aligned with results from human macrophage assays, where endosymbiont-containing spores protected the fungus from phagocytosis. Similarly, Richter et al. demonstrated that co-culturing the amoeba *Prostelium aurantium* with *Rhizopus* spores, hosting rhizoxin producing bacteria, resulted in detrimental effects of the toxin onto the amoeba, ultimately providing protection to the fungus (Richter et al., 2022). Whether virulence factors of other mucorales, such as those inducing apoptosis in macrophages as observed by López-Muñoz et al. for *M. circinelloides*, originated from interactions between amoeba and this *Mucor* species requires verification in an amoeba model (López-Muñoz et al., 2018).

## 6 Summary and outlook

As described in this review, the different well-established alternative models greatly facilitate compiling data to enhance our understanding of mucormycosis. The types of experiments performed and their significance for potential clinical applications are summarized in **Table 1**. All models comprise advantages and disadvantages (summarized in **Table 2**), which mostly hold true for investigating other fungal pathogens as well. The lack of standardized procedures and protocols and the lack of mimicking other forms of disease than the disseminated one are the most prominent. Besides those limitations, the beneficial nature of the presented models for pre-screening and evaluation of large strain sets or high number of antifungal agents, is especially important to meet the great variability seen in mucormycosis and,on long track, helps to minimize mammalians needed in follow-up experiments. To incorporate translational aspects, like the potential of diagnosing pulmonary mucormycosis via a breath test, or the efficacy of novel antifungal drugs, data for all clinically relevant Mucorales species will be needed, an aim that can easier be met with alternative model systems. Enhancing transcriptomics and proteomics studies of the individual model challenged with Mucorales, will greatly advance the suitability and comparability of the respective system. Following the principle of the 3 Rs (replacement, reduction, and refinement), replacing animal models for studying mucormycosis with more advanced cell-culture based methods has not yet been exhausted fully, which holds promising applications for advancing mucormycosis research in the future.

**Table 1:** Type of experiments performed in mucormycosis and their potential translational application.

Model organism	Tests performed in mucormycosis studies (unique for mucormycosis in bold)	Translational aspects
*C. elegans*	• Study of virulence factors (Arf and Arf-like proteins, fermentative metabolism, siderophore production) • Impact of growth medium on virulence • **Impact of endosymbionts/their products on virulence**	• Identification of potential new drug targets • Control of serum iron levels in patients • Targeted antibiotic use against endosymbiont-carrying strains
*D. melanogaster*	• Study of virulence factors (CotH-genes, iron-metabolism, **tornadic-sheer stress**, VOCs)	• Identification of potential new drug targets • Control of serum iron levels in patients • Use of calcineurin inhibitors in traumatic wounds • Use of VOCs in patients’ breath for diagnosis
*G. mellonella*	• Study of virulence factors (CotH-genes, growth speed, myosin genes, oxidative stress tolerance, siderophore production, spore size, spore surface composition) • Testing of (new) antifungals and combination therapies • Impact of growth medium on virulence (iron concentration) • Impact of incubation temperature • Screening of virulence potential of different strains	• Identification of potential new drug targets • Control of serum iron levels in patients • Synergetic effects of drugs in antifungal therapy • Identification of new virulence traits as putative novel drug targets • Awareness of changes in epidemiology
*B. mori*	• Testing of new antifungals • Screening of virulence potential of environmental derived strains	• Potential new antifungals substances • Identification of potentially pathogenic species (awareness in diagnostic)
*D. rerio*	• Study of virulence factors (spore size) • Study of different infections sites • Study of phagocyte recruitment • **Impact of endosymbionts** • **Impact of microbial interactions with *P. aeruginosa* in traumatic wounds**	• Identification of potential new drug targets • Identification of strains with clinical relevance • Manifestation of diseases depending on tissue type • Antifungal prophylaxis in patients with neutropenia • Targeted antibiotic use against endosymbiont-carrying strains • Cautious use of antibiotics in traumatic wounds with potential fungal infections
Chicken egg	• Screening of virulence potential of different strains and their virulence traits	• Identification of potential new drug targets • Identification of strains with clinical relevance and correlation to origin
Amoeba	• **Impact of endosymbionts in evasion of phagocytosis**	• Targeted antibiotic use against endosymbiont-carrying strains

**Table 2:** Comparison of the alternative model organisms with focus on mucormycosis.

	*Caenorhabditis elegans*	*Drosophila melanogaster*	*Galleria mellonella*	*Bombyx mori*	*Danio rerio*	Chicken egg	Amoeba
General characteristics							
- Breeding	Yes	Yes	Yes	Yes	Yes	Yes	Yes
- costs	Very low	Reasonable	Very low	Very low	Reasonable	Low	Very low
- commercial availability	No	Yes	Yes	Yes	Yes	Yes	No
- Ethics approval necessary	No	No	No	No	Yes^1^	Yes^2^	No
- Temperature range	15-25°C	12-30°C	20-42°C	up to 37°C	28-28.5°C	37°C	22-28°C^3^
- Genetic manipulation	Possible	Possible	Not possible	Possible	Possible	Not possible	Possible
- Gene homology to humans	65%	60%	n. d.	58%	70%	75%	n. d.
Experimental handling							
- Ease of handling	Easy	Special training and facilities needed	Very easy	Very easy	Special training and facilities needed	Very easy	Very easy
- Injection of defined inoculum	Not possible	Not possible	Possible	Possible	Possible^1^	Possible	Not possible
- Multiple injections without harm	No injection	Not possible	Possible	Possible	Possible^1^	Possible	No injection
- Dissection of organs	Not possible	Not possible	Possible	Possible	Possible^1^	Possible	Not possible
- Different infection routes	Not possible	Not possible	Not possible	Possible	Possible^1^	Possible	Not possible
- Transcriptomics/proteomics	Yes	Yes	Yes	Yes	Yes	n. d.	Yes
Evaluation of antifungal agents	Yes	Yes	Yes	Yes	Yes	Yes	Yes
- Tested against Mucorales	No	Yes	Yes	Yes	No	No	No
Correlation of virulence potential to mammals	Yes	Yes	Yes	Yes	Yes	Yes	Yes
Immunology							
- Similarities in innate immunity	Yes	Yes	Yes	Yes	Yes	Yes	Yes
- adaptive immunity & cytokines	No	No	No	No	Yes^1^	Yes^2^	No
- *Ex vivo* studies with immune cells	Not possible	Possible	Possible	Possible	Possible	Not possible	Not possible
- Induction of immunosuppression	Possible	Possible	n. d.	Possible	Possible	n. d.	Not possible

^1^for adult zebrafish.

^2^by the 18^th^ day.

^3^depending on species.

## Author contributions

JS: Writing – original draft, Writing – review & editing. UB: Conceptualization, Writing – original draft, Writing – review & editing.
